# A Study of the Mechanical Behaviour of Boron Nitride Nanosheets Using Numerical Simulation

**DOI:** 10.3390/nano13202759

**Published:** 2023-10-13

**Authors:** Nataliya A. Sakharova, André F. G. Pereira, Jorge M. Antunes

**Affiliations:** 1 Centre for Mechanical Engineering, Materials and Processes (CEMMPRE), Advanced Production and Intelligent Systems, Associated Laboratory (ARISE), Department of Mechanical Engineering, University of Coimbra, Rua Luís Reis Santos, Pinhal de Marrocos, 3030-788 Coimbra, Portugal; andre.pereira@uc.pt (A.F.G.P.); jorge.antunes@dem.uc.pt (J.M.A.); 2 Abrantes High School of Technology, Polytechnic Institute of Tomar, Quinta do Contador, Estrada da Serra, 2300-313 Tomar, Portugal

**Keywords:** boron nitride, nanosheets, elastic properties, modeling, numerical simulation

## Abstract

Hexagonal boron nitride (h-BN) nanosheets are attractive materials for various applications that require efficient heat transfer, surface adsorption capability, biocompatibility, and flexibility, such as optoelectronics and power electronics devices, nanoelectromechanical systems, and aerospace industry. Knowledge of the mechanical behavior of boron nitride nanosheets is necessary to achieve accurate design and optimal performance of h-BN-based nanodevices and nanosystems. In this context, the Young’s and shear moduli and Poisson’s ratio of square and rectangular boron nitride nanosheets were evaluated using the nanoscale continuum modeling approach, also known as molecular structural mechanics. The latter allows robust and rapid assessment of the elastic constants of nanostructures with graphene-like lattices. To date, there is a lack of systematic research regarding the influence of input parameters for numerical simulation, loading conditions, size, and aspect ratio on the elastic properties of the h-BN nanosheets. The current study contributes to filling this gap. The results allow, on the one hand, to point out the input parameters that lead to better agreement with those available in the literature. On the other hand, the Young’s and shear moduli, and Poisson’s ratio calculated in the present work contribute to a benchmark for the evaluation of elastic constants of h-BN nanosheets using theoretical methods.

## 1. Introduction

**Figure 1:**
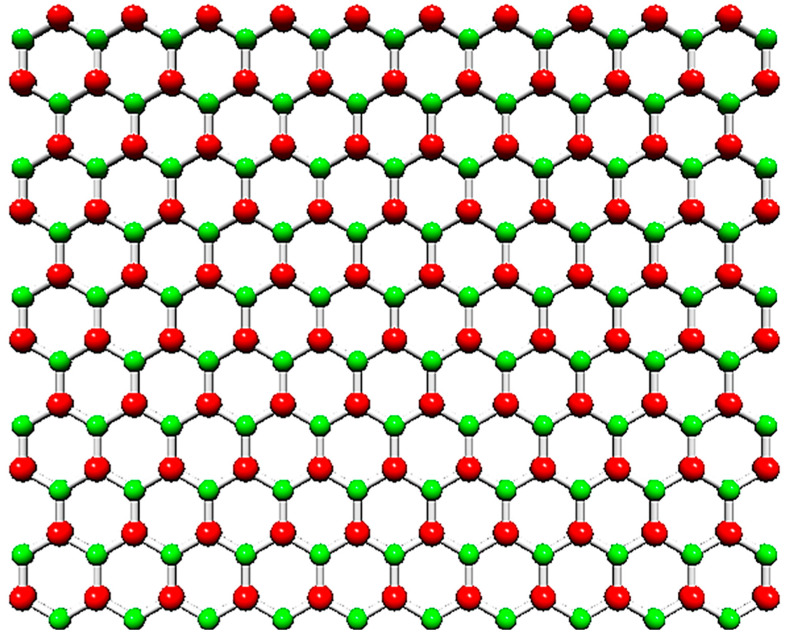
Hexagonal boron nitride (h-BN) nanosheet. The B atoms are shown in red; the N atoms are in green.

Nanosheets of hexagonal boron nitride (h-BN) are two-dimensional (2D) structures with honeycomb arrangement, which have remarkable physical, chemical, and mechanical properties [1,2,3,4]. The schematic representation of the graphene-like boron nitride (BN) nanosheet is shown in Figure 1. Alternating boron (B) and nitrogen (N) atoms of the h-BN lattice are connected by covalent sp^2^—bonds, which results in resemblances of the structure of boron nitride nanosheet with that of 2D graphene.

On the one hand, this makes h-BN nanostructure a good candidate to replace graphene in various applications, including high-quality electronics [5] and reinforced nanocomposites [6]. On the other hand, it allows the development of novel heterostructures and devices, taking advantage of the h-BN properties that are different from those of graphene [7,8]. Moreover, 2D boron nitride is established as a promising material to be employed in resonant nanoelectromechanical systems (NEMS) [9], membranes for toxic gases separation [10] and water purification [11], electrodes for Mg-ion batteries [12], and thermally conductive heat spreaders [13].

In view of its promising applications, it is necessary to develop a straightforward, controllable, and accessible method for the synthesis and mass production of h-BN nanosheets (NSs). To date, two techniques have been commonly used to prepare boron nitride nanosheets (BNNSs): the first utilizes chemical vapor deposition (CVD) [4,14,15,16], and the second allows to obtain nanosheets by exfoliation from bulk boron nitride [1,5,6,17,18]. An et al. [18] recently developed an innovative and ecologically friendly technique to obtain high-quality BNNSs by exfoliation, which allows them to be produced in mass quantity and controls their thickness. Furthermore, a simple and low-cost process for chemical synthesis of single-layer BNNSs that uses three different boric acids as precursors was recently proposed [19]. Li et al. [20] used solid-state borates as precursors within a borate nitridation reaction for efficient and low-cost mass fabrication of BNNSs.

Knowledge about the mechanical behavior of boron nitride NSs is important not only for designing efficient and robust nanosystems and nanodevices, but also for understanding how the mechanical behavior can influence the electronic, optical, and chemical properties of 2D h-BN. Nevertheless, the studies on the mechanical properties of BNNSs remain insufficient. Investigations dedicated to the evaluation of the mechanical properties of BNNSs are divided into theoretical and experimental. Theoretical (analytical and numerical) works, which are the majority, are mainly based on atomistic and nanoscale continuum modeling approaches. The atomistic approach consists of using ab initio density functional theory (DFT) calculations and molecular dynamics (MD). When atomistic modeling is employed to assess the elastic properties of BNNSs, the choice of potential functions for describing the interactions between boron (B) and nitride (N) atoms in the nanosheet influences the results. Kudin et al. [21], Wu et al. [22], Ahangari et al. [23], and Peng et al. [24] used ab initio DFT calculations to study the elastic properties of BNNSs. Mirnezhad et al. [25] applied DFT combined with quasi-harmonic approximation (QHA) to evaluate the Young’s modulus of BNNSs. With the same objective, Le [26], Zhao and Xue [27], Han et al. [28], Verma et al. [29], Los et al. [30], Thomas et al. [31], Salvati et al. [32], Eshkalak et al. [33], Mortazavi and Rémond [34], and Vijayaraghavan and Zhang [35] employed MD simulations with Tersoff and Tersoff-like potentials to describe the interactions between B and N atoms. Using MD simulation combined with atomic force microscopy (AFM), Qu et al. [36] assessed the bending and interlayer shear elastic moduli of multilayered boron nitride NSs. The nanoscale continuum modeling (NCM), also known as the molecular structural mechanics (MSM) approach makes use of the linking between the molecular structure of the nanosheet and solid mechanics, considering the bonds between B and N atoms as elastic elements, such as springs or beams. The main challenge in applying the NCM/MSM approach is an appropriate choice of the force field constants to simulate the bond between the two atoms, B and N, in the diatomic nanostructure. Le and Nguyen [37] used a global stiffness matrix assembled with element stiffness matrices in their finite element (FE) study within the framework of the NCM/MSM approach, to evaluate the Young’s and shear moduli, and Poisson’s ratio of BNNSs. With the same purpose, Georgantzinos et al. [38] represented the B–N bonds as spring elements in the FE model for BNNS, in their NCM/MSM study. Tapia et al. [39] also used the NCM/MSM approach to evaluate the Young’s and shear moduli and Poisson’s ratio of BNNSs, but the B–N bond was replaced by a beam element. Qin et al. [40] modeled the B–N bond as a beam element as well and calculated the Young’s modulus of BNNSs, using a closed-form solution. Ansari et al. [41] derived analytical expressions with the NCM/MSM approach to assess the Young’s modulus and Poisson’s ratio of BNNSs. It is worth noting that among the aforementioned studies, only Georgantzinos et al. [38] and Tapia et al. [39] investigated the elastic properties of rectangular BN nanosheets, i.e., nanoribbons. In ab initio DFT and MD simulations, there is ambiguity in the selection of functionals of electronic density and potential functions to describe interatomic interactions, respectively, which leads to a considerable scattering of the elastic properties evaluated in the abovementioned theoretical works. In the case of the NCM/MSM approach, this scattering is related to the choice of the force field constants and the type and geometry of the elastic element to represent the B–N bond.

With regard to experimental studies on the determination of the elastic properties of the boron nitride nanosheets, Bosak et al. [42], to the best of our knowledge, were the first to measure the Young’s modulus of h-BN, making use of inelastic X-ray scattering. Then, Song et al. [14] determined the Young’s modulus of BNNSs composed of two to five layers, with the aid of nanoindentation tests performed using AFM. The AFM technique was also used by Kim et al. [43] to carry out nanoindentation tests to determine the Young’s modulus of multilayer h-BN. Falin et al. [6] evaluated the Young’s modulus of single-layer and multilayer (up to 8 layers) BNNSs by nanoindentation, using AFM.

The present study aims to assess the elastic moduli (Young’s and shear) and Poisson’s ratio of one-layer square and rectangular boron nitride nanosheets (BNNSs). The rectangular BNNSs are usually designated as boron nitride nanoribbons (BNNRs) and have merited less research attention so far. To this end, the B–N interatomic bonds were simulated as equivalent beams under the NCM/MSM approach, and three-dimensional (3D) FE models of square and rectangular NSs were built. The mechanical response of the BNNSs and BNNRs was studied under numerical in-plane tensile and shear tests. The influence of the nanosheet size and aspect ratio, loading case, and input parameters for numerical simulation, on the elastic properties of the BN square and rectangular NSs was comprehensively investigated, turning the present work into a systematic study that has been lacking so far.

## 2. Materials and Methods

### 2.1. Geometrical Characteristics of BNNSs and BNNRs

Single-layer square boron nitride nanosheets with four different sizes, as shown in Table 1, were studied. The sizes of the rectangular nanosheets (NSs) with different aspect ratios, which make up four nanoribbons (NRs), are also shown in Table 1.

The finite element meshes of BNNSs and BNNRs were obtained using the Nanotube Modeler© software (version 1.8.0, ©JCrystalSoft), which produces the Program Database files. These files were later converted to a format compatible with commercial codes for finite element analysis (FEA), using the in-house application InterfaceNanosheets.NS.

**Figure 2:**
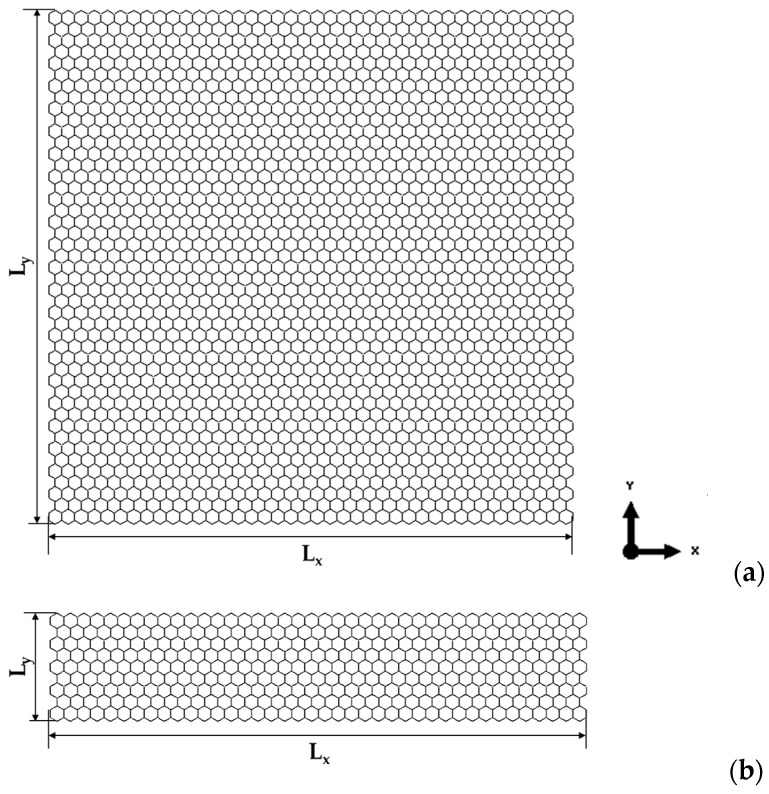
Geometrical parameters of (**a**) BN nanosheet (S3) and (**b**) BN nanoribbon (R2).

Examples of FE meshes of BN nanosheet (S3) and BN nanoribbon (R2) are shown in Figure 2, along with the geometrical parameters of NS and NR.

### 2.2. Input Parameters for the FE Models of BNNSs and BNNRs

In the present study, the NCM/MSM method was used, which replaced the B–N bonds of boron nitride nanostructures with equivalent beams. The equivalent continuum structure comprised of elastic beam elements can be correlated with the molecular structure, characterized by the following force constants: the bond stretching, $k_{r}$; bond bending, $k_{\theta }$; and torsional resistance, $k_{\tau }$. In this way, the relationships between the tensile, $E_{b}A_{b}$, bending, $E_{b}I_{b}$, and torsional, $G_{b}J_{b}$, rigidities of beams with length *l*, and $k_{r}$, $k_{\theta }$ and $k_{\tau }$ force constants are the basis for the analysis of the mechanical response of BNNSs and BNNRs [44]:

(1) $$E_{b}A_{b}=lk_{r},\;E_{b}I_{b}=lk_{\theta },\;G_{b}J_{b}=lk_{\tau }.$$

In Equation (1), $A_{b}$ is the cross-section area, $I_{b}$ is the moment of inertia, and $J_{b}$ is the polar moment of inertia of the beam; these are given as follows in the case of a circular cross-section area:

(2) $$A_{b}=\pi d^{2}/4,\;I_{b}=\pi d^{4}/64,\;J_{b}=\pi d^{4}/32,$$

where d is the diameter of the beam element.

The input parameters for the numerical simulation (i.e., the Young’s, $E_{b}$, and shear, $G_{b}$, moduli of the beam) are calculated from Equation (1), knowing the values of the force constants $k_{r}$, $k_{\theta }$, and $k_{\tau }$. For boron nitride nanostructures, various force constant values have been reported in the literature, for example, as analyzed in the work of Sakharova et al. [45]. The dissimilarities in the values of $k_{r}$, $k_{\theta }$, and $k_{\tau }$ can be attributed to the approach chosen for their calculation. Two approaches, both commonly accepted by the research community, were considered for the calculation of the bond stretching, $k_{r}$, and the bond bending, $k_{\theta }$, force constants. Firstly, $k_{r}$, and $k_{\theta }$ force field constants of BN nanostructures were calculated based on universal force fields (UFFs) [46], where the force field parameters were evaluated using general rules based on the element and its connectivity. The bond stretching, $k_{r}$, and bond bending, $k_{\theta }$, force constants are expressed in the UFF method as follows:

(3) $$k_{r}=664.12\frac{Z_{1}^{*}Z_{2}^{*}}{a_{B–N}^{3}},$$

(4) $$k_{\theta }=830.15\frac{Z_{1}^{*}Z_{2}^{*}}{{\left(\sqrt[{}]{3}a_{B–N}\right)}^{3}},$$

where $Z_{1}^{*}$ and $Z_{2}^{*}$ are the effective charges of the B and N atoms, respectively, and $a_{B–N}$ is the equilibrium length of the B–N covalent bond. The values of $a_{B–N}$ = 0.145 nm [47], $Z_{1}^{*}=$ 1.755 charge for B atom [46], and $Z_{2}^{*}$ = 2.544 charge for N atom [46], were considered in the current study.

Next, another approach was also used to determine $k_{r}$ and $k_{\theta }$ for BN nanostructure, which uses ab initio DFT calculations combined with analytical relationships, deriving from molecular mechanics (MM). This DFT + MM method is based on the following MM expressions for the surface Young’s modulus, $E_{s}$, and the Poisson’s ratio, ν [48]:

(5) $$\left\{\begin{array}{c} E_{s}=\frac{4\sqrt{3}k_{r}k_{\theta }}{k_{r}\frac{a_{B–N}^{2}}{2}+9k_{\theta }}\\ \nu =\frac{k_{r}a_{B–N}^{2}\text{–}6k_{\theta }}{k_{r}a_{B–N}^{2}+18k_{\theta }} \end{array}\right.$$

The relationships to calculate the bond stretching, $k_{r}$, and bond bending, $k_{\theta }$, force constants were obtained by solving the system of Equation (5), as follows:

(6) $$k_{r}=\frac{9E_{s}}{\sqrt{3}\left(1\text{–}\nu \right)},$$

(7) $$k_{\theta }=\frac{E_{s}a_{B–N}^{2}}{2\sqrt{3}\left(1+3\nu \right)},$$

where $E_{s}$ and ν are the surface Young’s modulus and Poisson’s ratio, respectively, of the BN nanosheet, and $a_{B–N}$ is the bond length. The values of $E_{s}$ = 267 nN/nm and ν = 0.21 used in Equations (5) and (6) were obtained from the results of the DFT calculations by Şahin et al. [47], who employed first-principles plane-wave calculations within DFT for total energy to this end.

Lastly, the torsion resistance constant, $k_{\tau }$, was obtained from the work of Ansari et al. [49], who calculated the connection of the bending rigidity of BN nanosheet, D, and the value of $k_{\tau }$, using the equation MM: $k_{\tau }$ = 24D. The authors also evaluated the BNNS bending rigidity, D, in their study [49], based on their own DFT calculations combined with the generalized gradient approximation (GGA) and using the Quantum-Espresso code.

Moreover, the bond stretching, $k_{r}$, bond bending, $k_{\theta }$, and torsional resistance, $k_{\tau }$, force constants, obtained by Tapia et al. [39]—using ab initio DFT computations, without resourcing to MM relationships—were considered in the current study to calculate the input parameters for numerical simulation. In their work, Tapia et al. [39] performed DFT calculations with GGA using the SIESTA code.

The diameter of the beam, d, and its Young’s, $E_{b}$, and shear, $G_{b}$, moduli, were deduced by combining Equations (1) and (2), as follows:

(8) $$d=4\sqrt{\frac{k_{\theta }}{k_{r}}},\;E_{b}=\frac{k_{r}^{2}l}{4\pi k_{\theta }},\;G_{b}=\frac{{k_{r}^{2}k}_{\tau }l}{8\pi k_{\theta }^{2}}.$$

The Poisson’s ratio of the beam element, $\nu_{b}$, is given by the following expression [48]:

(9) $$\nu_{b}=\frac{k_{r}l^{2}–\;6k_{\theta }}{k_{r}l^{2}+18k_{\theta }}.$$

The beam length, *l*, in the present model, is equal to the B–N bond length, *l* = $a_{B–N}$ = 0.145 nm [47].

The geometrical and elastic properties of the equivalent beam elements constitute the input parameters for numerical simulation as shown in Table 2. The use of three sets of the force field constants, assessed by different calculation methods, allowed covering a comprehensive range of beam characteristics.

### 2.3. Finite Element Analysis and Elastic Properties of BNNSs and BNNRs

**Figure 3:**
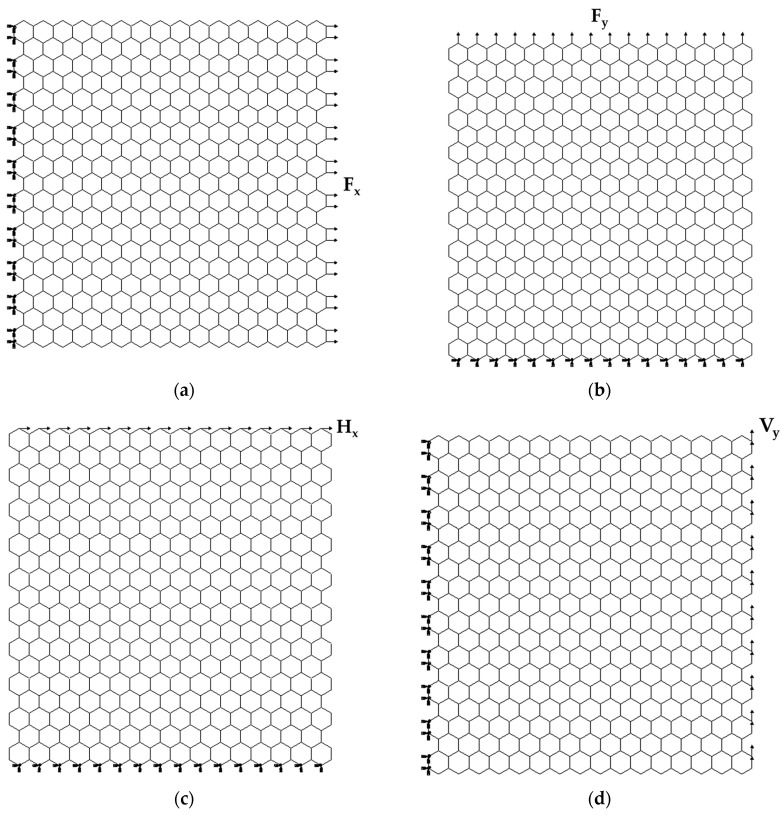
Schematic representation of the boundary and loading conditions for BN nanosheet S2: (**a**) tensile loading in the horizontal (zigzag) direction; (**b**) tensile loading in the vertical (armchair) direction, (**c**) in-plane shear loading in the horizontal direction; and (**d**) in-plane shear loading in the vertical direction.

The mechanical behavior of the BNNSs and BNNRs was studied under numerical tensile and in-plane shear tests, using the ABAQUS^®^ FE code. Figure 3 shows four studied loading cases with the respective boundary conditions for the case S2 (see Table 1) of BNNSs.

In the first loading case, shown in Figure 3a, the nodes of the left side of the BNNS were fixed, while an axial tensile force, $F_{x}$, was applied to the right (opposite) side. In the second case, the BNNS bottom side was fixed, and an axial force, $F_{y}$, was applied to the upper side nodes (see, Figure 3b). In the third loading case, represented in Figure 3c, the boundary conditions were the same as in the second case, while a shear force, $H_{x}$, was applied on the nodes of the upper side of the BNNS. In the fourth case, the boundary conditions were the same as in the first case, and a shear force, $V_{y}$, was applied to the edge nodes on the BNNS right side (see, Figure 3d). Therefore, according to the atomic arrangement of the nanosheet or nanoribbon along the horizontal and vertical directions, the zigzag and armchair configurations, respectively, of the BNNS (BNNR) are considered.

**Figure 4:**
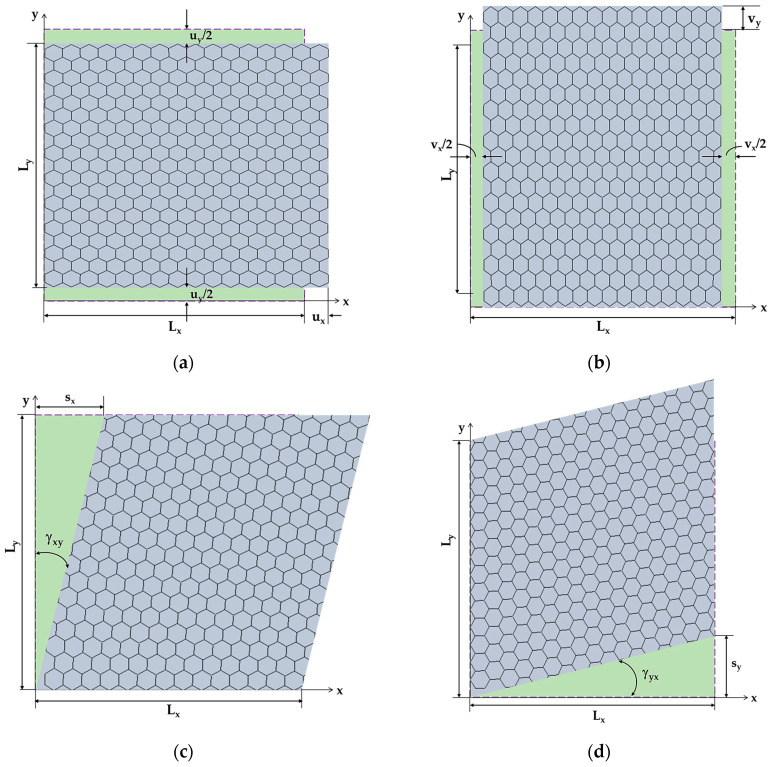
Schematic representation of the deformed (depicted in lilac) BN nanosheet S2: (**a**) axial displacement, $u_{x}$, and transversal displacement, $u_{y}$, of the BNNS under axial force, $F_{x}$; (**b**) axial displacement, $v_{x}$, and transversal displacement, $v_{y}$, of the BNNS under transversal force, $F_{y}$; (**c**) axial displacement, $s_{x}$, of the BNNS under in-plane shear load, $H_{x}$; and (**d**) transversal displacement, $s_{y}$, of the BNNS under in-plane shear load, $V_{y}$. The undeformed square NS is depicted in green.

The axial displacement, $u_{x}$, (corresponding to elongation in the x-direction) and transversal displacement, $u_{y}$, (corresponding to the contraction in the y-direction) under the axial tensile load $F_{x}$, were obtained from FEA (Figure 4a). Subsequently, the Young’s modulus along the *x*-axis, $E_{x}$, and the Poisson’s ratio, $\nu_{\mathrm{xy}}$, can be assessed by the following expressions, respectively [39]:

(10) $$E_{x}=\frac{F_{x}L_{x}}{u_{x}L_{y}t_{n}},$$

(11) $$\nu_{\mathrm{xy}}=\frac{u_{y}L_{x}}{{u_{x}L}_{y}},$$

where $L_{x}$ and $L_{y}$ are the BNNS side lengths (see, Figure 2); $t_{n}$ is the nanosheet thickness; the transversal displacement, $u_{y}$, is measured at $x=L_{x}/2$ (see, Figure 4a).

To calculate the Young’s modulus along the *y*-axis, $E_{y}$, and the Poisson’s ratio, $\nu_{yx}$, the displacements of the BNNS in the y-direction, $v_{y}$, and in the x-direction, $v_{x}$, under the applied load $F_{y}$, are taken from the FEA (Figure 4b). Consequently, the Young’s modulus along the *y*-axis, $E_{y}$, and the Poisson’s ratio, $\nu_{yx}$, were assessed as follows:

(12) $$E_{y}=\frac{F_{y}L_{y}}{v_{y}L_{x}t_{n}},$$

(13) $$\nu_{\mathrm{yx}}=\frac{v_{x}L_{y}}{v_{y}L_{x}},$$

with the axial displacement, $v_{x}$, measured at $y\;=L_{y}/2$ (see, Figure 4b).

The displacement of the BNNS in the x-direction, $s_{x}$, under the in-plane shear load $H_{x}$, was taken from the FEA to calculate the shear strain, $\gamma_{\mathrm{xy}}$ (Figure 4c). Then, the shear modulus, $G_{\mathrm{xy}}$, of the BNNS can be assessed by the following expression [39]:

(14) $$G_{\mathrm{xy}}=\frac{H_{x}}{\gamma_{\mathrm{xy}}L_{x}t_{n}},\;\gamma_{\mathrm{xy}}=\frac{s_{x}}{L_{y}}.$$

The shear modulus, $G_{\mathrm{yx}}$, of the BNNS is calculated as follows:

(15) $$G_{\mathrm{yx}}=\frac{V_{y}}{\gamma_{\mathrm{yx}}L_{y}t_{n}},\;\gamma_{\mathrm{yx}}=\frac{s_{y}}{L_{x}},$$

where $V_{y}$ is in-plane shear load, $s_{y}$ is the BNNS displacement in the y-direction, obtained from the FEA (see, Figure 4d), $\gamma_{\mathrm{yx}}$ is the shear strain, $L_{x}$ and $L_{y}$ are the NS side lengths, and $t_{n}$ is the NS thickness.

To calculate the $G_{\mathrm{xy}}$ and $G_{\mathrm{yx}}$ shear moduli, the respective displacements, $s_{x}$ and $s_{y}$ were measured in the central part of the NS to avoid effects at the edge nodes, where boundary and loading conditions were applied.

In the present study, the nanosheet thickness, $t_{n}$, was taken equal to 0.34 nm, which was the same as the interlayer space of graphene. Such value, which was experimentally confirmed using transmission electron microscopy (TEM), $t_{n}$ = 0.338 ± 0.004 nm [50], has been used by several researchers (see, for example [21,38,41]).

## 3. Results and Discussion

### 3.1. Effect of Size and Aspect Ratio on Elastic Properties of Boron Nitride Nanosheets

**Figure 5:**
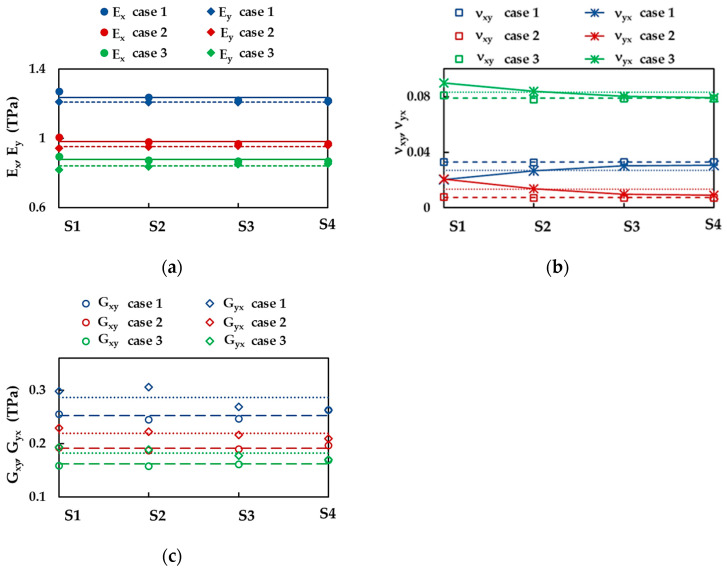
(**a**) Young’s moduli, $E_{x}$ (zigzag) and $E_{y}$ (armchair), (**b**) Poisson’s ratios, $\nu_{\mathrm{xy}}$ and $\nu_{yx}$, and (**c**) shear moduli, $G_{\mathrm{xy}}$ and $G_{\mathrm{yx}}$, of the BNNSs for the four sizes (Table 1) and the three cases of the input parameters (Table 2). The horizontal lines represent the average value of the respective Young’s modulus, Poisson’s ratio, and shear modulus.

Figure 5a shows the Young’s moduli along x-direction (zigzag), $E_{x}$, and y-direction (armchair), $E_{y}$, calculated by Equations (10) and (12), respectively, for the BN square nanosheets of different sizes (see Table 1), taking into consideration the three cases of input parameters for numerical simulation presented in Table 2. Both $E_{x}$ and $E_{y}$ moduli are nearly constant for all sizes of BNNSs studied—except the Young’s modulus for zigzag configuration, $E_{x}$, of the smallest nanosheet S1, for which a slight increase in the value of $E_{x}$ is observed—in the case 1, 2, and 3, of the input parameters. The average values of Young’s moduli, $E_{x}$ and $E_{y}$, represented in Figure 5a by solid and dashed lines, respectively, are considered in further analysis.

The results regarding the influence of the sheet size on the BNNS Poisson’s ratios, $\nu_{\mathrm{xy}}$ and $\nu_{\mathrm{yx}}$, evaluated by Equations (11) and (13), respectively, are presented in Figure 5b. The Poisson’s ratio, $\nu_{\mathrm{xy}}$,—corresponding to the NS contraction in the y-direction when the nanosheet elongates along the *x*-axis (zigzag direction)—has nearly the same value for all sizes of the BNNSs under study, regardless of the input parameter. Thus, hereinafter, the average value of $\nu_{\mathrm{xy}}$ (represented by dashed lines for each case 1, 2, and 3 in Figure 5b) is taken into consideration.

For cases 2 and 3 of the input parameters, the Poisson’s ratio, $\nu_{\mathrm{yx}}$—resultant of the NS contraction in the x-direction under the force applied along the *y*-axis (armchair direction)—slightly decreases with the NS size and inclines to the value close to that of $\nu_{\mathrm{xy}}$. On the contrary, for case 1, the $\nu_{\mathrm{yx}}$ value slightly increases with the NS size, and inclines to that of $\nu_{\mathrm{xy}}$. To simplify the analysis, the average values of the Poisson’s ratio, $\nu_{\mathrm{yx}}$, represented by dotted lines in Figure 5b, are considered henceforward.

Figure 5c shows the shear moduli for zigzag, $G_{\mathrm{xy}}$, and armchair $G_{\mathrm{yx}}$, nanosheet configurations, evaluated by Equations (14) and (15), respectively, of the BNNSs of different sizes from Table 1, for the three cases of the input parameters (Table 2). Both $G_{\mathrm{xy}}$ and $G_{\mathrm{yx}}$ moduli are approximately constant for all BNNS sizes studied, although the shear modulus, $G_{\mathrm{yx}}$, calculated for case 1, shows a greater scattering of the values obtained for different NS sizes. The dashed and dotted lines in Figure 5c correspond to the average values of $G_{\mathrm{xy}}$ and $G_{\mathrm{yx}}$, respectively, which are considered below.

The average values of the Young’s, $E_{x}$ and $E_{y}$, and shear, $G_{\mathrm{xy}}$ and $G_{\mathrm{yx}}$, moduli, and the Poisson’s ratio, $\nu_{\mathrm{xy}}$ and $\nu_{\mathrm{yx}}$, are summarized in Table 3 for the three cases of the input parameters.

**Figure 6:**
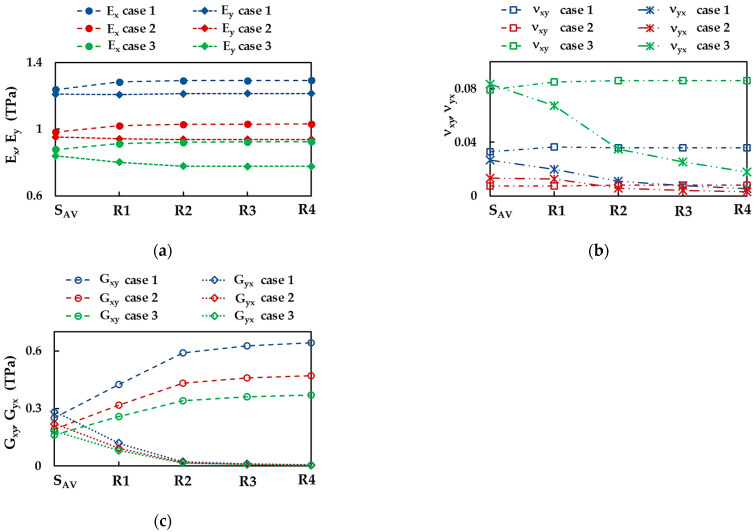
(**a**) Young’s moduli, $E_{x}$ (zigzag) and $E_{y}$ (armchair), (**b**) Poisson’s ratios, $\nu_{\mathrm{xy}}$ and $\nu_{yx}$ and (**c**) shear moduli $G_{\mathrm{xy}}$ and $G_{\mathrm{yx}}$ of the four nanoribbons R1–R4 (Table 1) and BNNS (the average values from Table 3), and the three cases of the input parameters (Table 2).

To study the influence of the aspect ratio on the elastic properties of the BNNS nanoribbons, the Young’s moduli, $E_{x}$ and $E_{y}$, the Poisson’s ratios, $\nu_{\mathrm{xy}}$ and $\nu_{\mathrm{yx}}$, and the shear moduli, $G_{\mathrm{xy}}$ and $G_{\mathrm{yx}}$, were plotted in Figure 6a–c, respectively, for the four nanorribons R1–R4 (see, Table 1) and the nanosheets (the average values from Table 3), taking into consideration the three cases of the input parameters.

The Young’s modulus in zigzag direction, $E_{x}$, increases from nanosheet to nanoribbon R1, and subsequently, with increasing aspect ratio, $E_{x}$ takes a stable value, depending on the case of the input parameters (see, Figure 6a). The stable values of $E_{x}$, evaluated for the BNNRs, are 1.293 TPa, 1.032 TPa, and 0.925 TPa for cases 1, 2, and 3, respectively, which are slightly higher than $E_{x}$ obtained for BNNS. For case 1, the BNNRs Young’s modulus in armchair direction, $E_{y}$, remains nearly constant with increasing aspect ratio and its value is equal to that of BNNSs. Regarding the two other cases of the input parameters, the value of $E_{y}$ decreases from NS to nanoribbon R2, and then becomes stable, with values of 0.938 TPa and 0.777 TPa for cases 2 and 3, respectively, corresponding to that of the nanoribbons with higher aspect ratios, R3 and R4.

The Poisson’s ratio, $\nu_{\mathrm{xy}}$, slightly increases when moving from NS to NRs and inclines to the same value, $\nu_{\mathrm{xy}}$ = 0.036, 0.008, 0.086 for cases 1, 2, and 3, respectively, with increasing aspect ratio (see, Figure 6b). On the other hand, the Poisson’s ratio, $\nu_{\mathrm{yx}}$, decreases from the value for NS to that of nanoribbon R4, with a greater decreasing rate for NRs, which have lower aspect ratios. For the NR with an aspect ratio of 1:10 (R4), $\nu_{\mathrm{yx}}$ = 0.005, 0.003, 0.018 for cases 1, 2, and 3, respectively, whose values are about 4.7 times lower than those calculated for NS.

The shear modulus, $G_{\mathrm{xy}}$, increases at the transition from nanosheet to nanoribbon R2, and then $G_{\mathrm{xy}}$ stabilizes and takes values that are at about 2.4 higher than those for NS, viz. 0.643 TPa, 0.472 TPa, and 0.370 TPa for cases 1, 2, and 3, respectively (see, Figure 6c). On the contrary, the shear modulus, $G_{\mathrm{yx}}$, decreases with increasing aspect ratio, and the decreasing rate is greater for BNNRs with smaller aspect ratios (up to R2).

### 3.2. Young’s Moduli and Poisson’s Ratio of BNNSs and BNNRs

**Figure 7:**
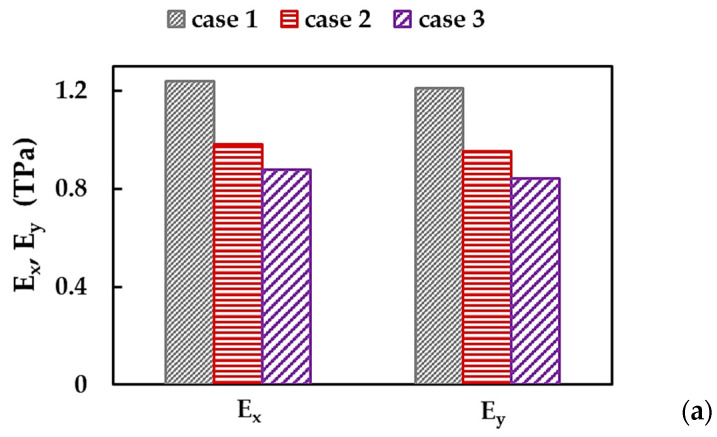

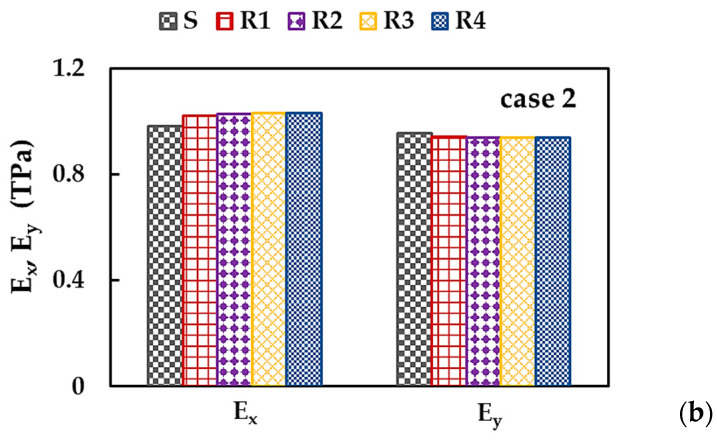
Young’s moduli, $E_{x}$ (zigzag direction) and $E_{y}$ (armchair direction) of (**a**) BNNSs for the three cases of the input parameters; and (**b**) BNNRs and BNNS for case 2.

Figure 7a shows the Young’s moduli of the BNNSs, along the zigzag direction, $E_{x}$, and the armchair direction, $E_{y}$, calculated by respective Equations (10) and (12) for the three cases of the input parameters. Both Young’s moduli, $E_{x}$ and $E_{y}$, evaluated for case 1 (UFF) are about 26.4% and 42.5% higher than those obtained for case 2 (DFT + MM) and case 3 (DFT), respectively. With regard to the BNNRs, their Young’s moduli, $E_{x}$ and $E_{y}$, are shown in Figure 7b together with those evaluated for the BNNSs, in case 2 of the input parameters. The value of $E_{x}$ (zigzag direction) is almost constant for all nanoribbons R1–R4 studied and 4.4% higher than that evaluated for the nanosheet. The Young’s moduli $E_{y}$ (armchair direction) of the BNNRs studied are nearly equal and correspond to the $E_{y}$ value for the BNNSs.

**Figure 8:**
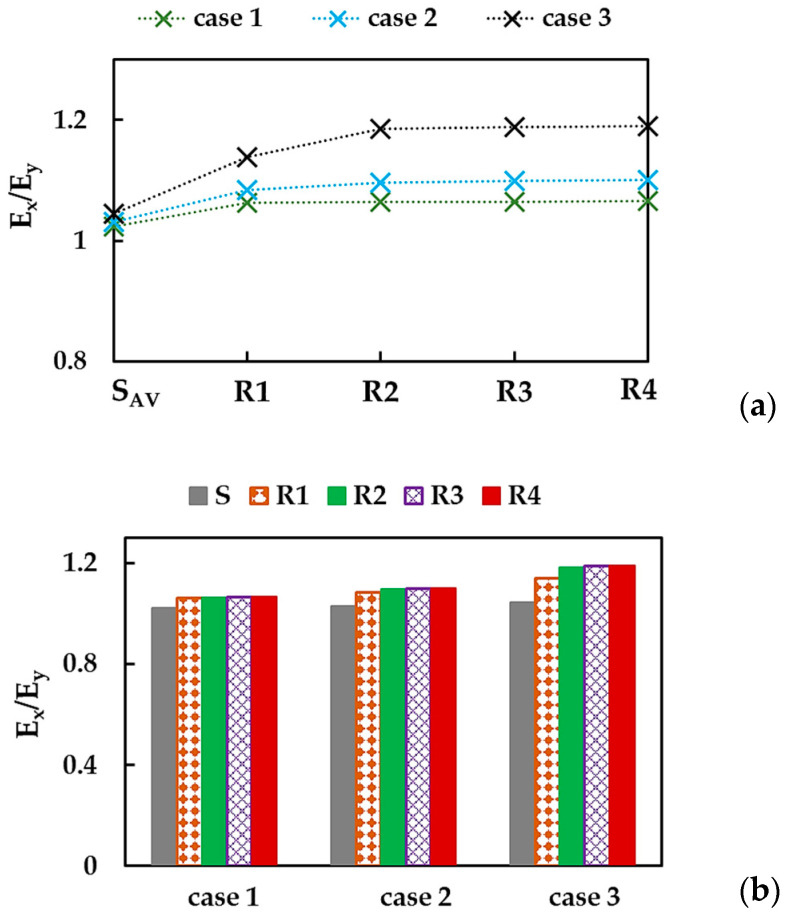
Evolutions of the ratio between the Young’s moduli in zigzag and armchair directions, $E_{x}$/$E_{y}$, for BNNSs and BNNRs, with (**a**) the NR aspect ratio and (**b**) the case of the input parameters.

As observed in Figure 7a,b, the BNNSs and BNNRs are not transversely isotropic, i.e., the Young’s modulus in the zigzag direction is higher than that in the armchair direction, $E_{x}$ > $E_{y}$. The ratio of $E_{x}$/$E_{y}$ for BN nanosheet and nanoribbons R1–R4 is shown in Figure 8a, considering the three cases of the input parameters. The value of $E_{x}$/$E_{y}$ for BNNS increases with the aspect ratio, which subsequently stabilizes, regardless of case 1, 2, or 3. The same results shown in Figure 8a, were plotted in Figure 8b—highlighting the case of input parameter—to simplify understanding. For BNNSs, the $E_{x}$/$E_{y}$ value for case 1 (UFF) is ≈1.02, which is about 0.8% and 2.0% lower than those for cases 2 (DFT + MM) and 3 (DFT), respectively. For the BNNRs, the stabilized ratio of $E_{x}$/$E_{y}$ ≈ 1.06 was obtained for case 1, which is nearly 3.1% and 10.5% lower than the respective values for cases 2 and 3.

**Figure 9:**
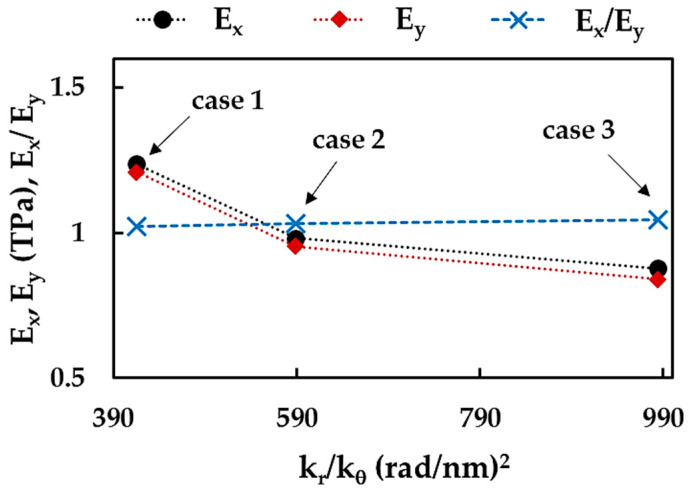
Evolutions of the BNNSs Young’s moduli, $E_{x}$ and $E_{y}$, and their relationship, $E_{x}$/$E_{y}$, with the ratio of the bond stretching and bond bending force constants, $k_{r}$/$k_{\theta }$.

To clarify the influence of the numerical simulation input parameters on the Young’s moduli of the BNNSs—along the zigzag and armchair directions, and their relationship, the values of $E_{x}$, $E_{y}$ and $E_{x}$/$E_{y}$—were plotted in Figure 9 as a function of the ratio between the bond stretching and bond bending force constants, $k_{r}$/$k_{\theta }$. The ratio, $k_{r}$/$k_{\theta }$, was chosen for this purpose because it is required to calculate the input parameters (see Equation (8)). The Young’s moduli, $E_{x}$ and $E_{y}$, decrease with the increase of the $k_{r}$/$k_{\theta }$ ratio, and the greatest decreasing rate is observed when moving from case 1 to case 2. The ratio between the Young’s moduli $E_{x}$/$E_{y}$ increases with the increase of $k_{r}$/$k_{\theta }$, viz. from case 1 to 3.

**Figure 10:**
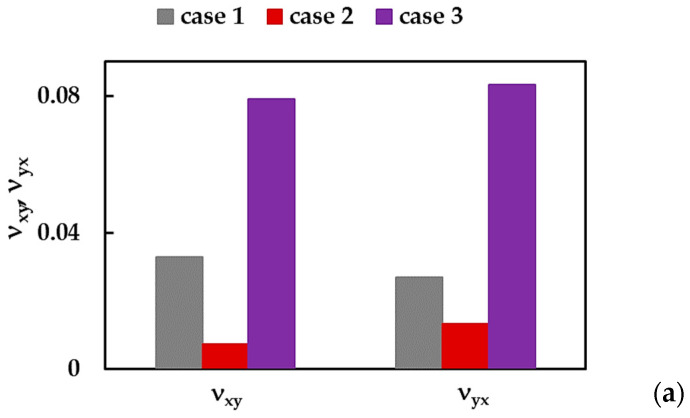

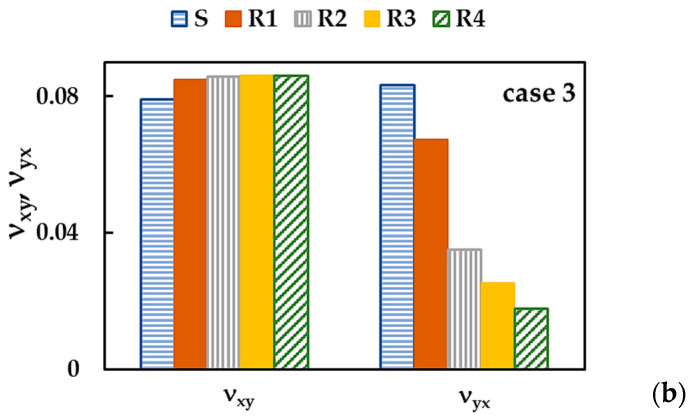
Poisson’s ratios, $\nu_{\mathrm{xy}}$ and $\nu_{\mathrm{yx}}$, of (**a**) BNNSs for the three cases of the input parameters; (**b**) BNNRs and BNNS for case 3.

Figure 10a shows the BNNSs Poisson’s ratios for zigzag, $\nu_{\mathrm{xy}}$, and armchair, $\nu_{\mathrm{yx}}$, orientations, calculated by Equations (11) and (13), respectively, for cases 1, 2, and 3 of the input parameters. The Poisson’s ratio, $\nu_{\mathrm{xy}}$, evaluated for case 1 (UFF) is about 4.5 times higher and 2.4 times lower than the $\nu_{\mathrm{xy}}$ obtained considering case 2 (DFT + MM) and 3 (DFT), respectively. The value of $\nu_{\mathrm{yx}}$ for case 1 is about 2 times higher when compared to that for case 2, and about 3 times lower than $\nu_{\mathrm{yx}}$, for case 3. The influence of the NS aspect ratio on the Poisson’s ratios, $\nu_{\mathrm{xy}}$ and $\nu_{\mathrm{yx}}$, of the BNNS and BNNRs is analyzed in Figure 10b for case 3.

The Poisson’s ratio, $\nu_{\mathrm{xy}}$, increases in the transition from BNNS to BNNRs, and becomes stable as nanoribbon attains $L_{y}{:L}_{x}$ = 1:5 (R2). On the contrary, the Poisson’s ratio $\nu_{\mathrm{yx}}$, gradually decreases with increasing the NS aspect ratio.

**Figure 11:**
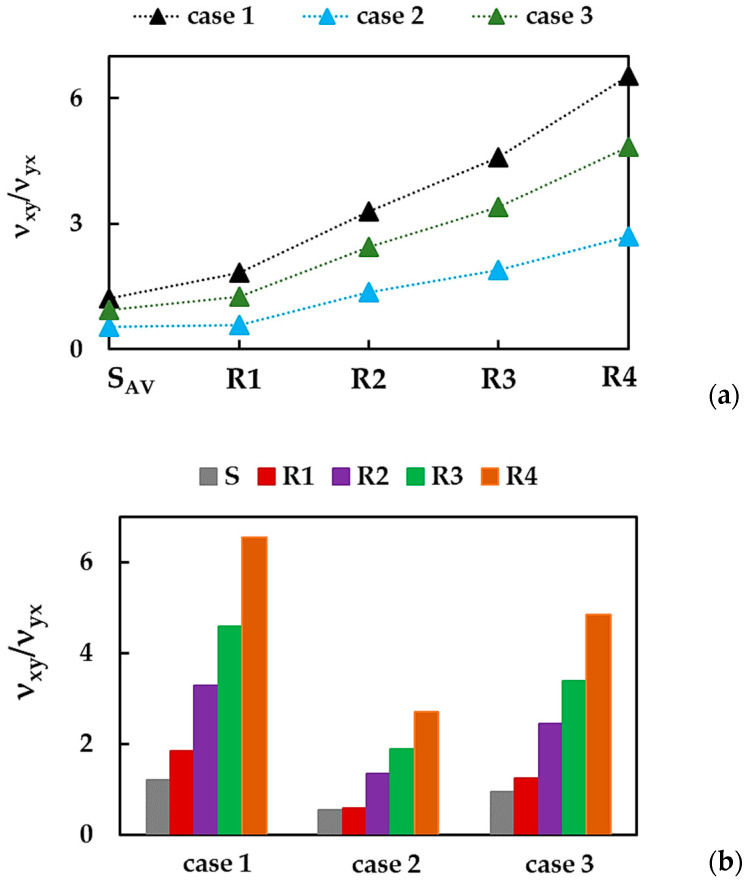
Evolutions of the ratio, $\nu_{\mathrm{xy}}$/$\nu_{\mathrm{yx}}$, for BNNSs and BNNRs, with (**a**) the NS aspect ratio and (**b**) the case of the input parameters.

The ratio of $\nu_{\mathrm{xy}}$/$\nu_{\mathrm{yx}}$ for the BNNSs and the BNNRs is presented in Figure 11a,b for cases 1, 2, and 3 of the input parameters.

The relationship between Poisson’s ratios—calculated when the NS (NR) shrinks in the y-direction, $\nu_{\mathrm{xy}}$, and in the x-direction, $\nu_{\mathrm{yx}}$—increases with the increase in the NR width (the edge length, $L_{x}$), i.e., from $L_{y}{:L}_{x}$ = 1:1 (nanosheet) to 1:10 (nanoribbon R4), in the case 1, 2, and 3 of the input parameters. The value of $\nu_{\mathrm{xy}}$/$\nu_{\mathrm{yx}}$ for case 1 is higher when compared to that in case 3, which, in turn, is higher than that of $\nu_{\mathrm{xy}}$/$\nu_{\mathrm{yx}}$ for case 2.

**Figure 12:**
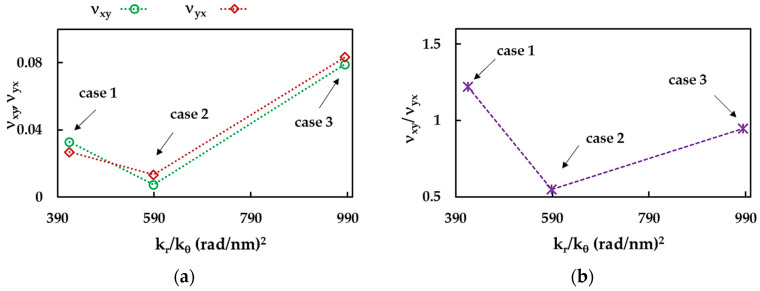
Evolutions of (**a**) the Poisson’s ratios, $\nu_{\mathrm{xy}}$ and $\nu_{\mathrm{yx}}$, and (**b**) their relationship, $\nu_{\mathrm{xy}}$/$\nu_{\mathrm{yx}}$, as a function of the ratio between the bond stretching and bond bending force constants, $k_{r}$/$k_{\theta }$, for the BNNSs.

To study the influence of the input parameters, the Poisson’s ratios, $\nu_{\mathrm{xy}}$ and $\nu_{\mathrm{yx}}$, and their relationship, $\nu_{\mathrm{xy}}$/$\nu_{\mathrm{yx}}$, for the BNNSs were plotted as a function of the ratio between the force constants, $k_{r}$/$k_{\theta }$, in Figure 12a,b. All values, $\nu_{\mathrm{xy}}$, $\nu_{\mathrm{yx}}$, and $\nu_{\mathrm{xy}}$/$\nu_{\mathrm{yx}}$, decrease with the increase in the ratio $k_{r}$/$k_{\theta }$, from case 1 to case 2, and then increase with further increase in $k_{r}$/$k_{\theta }$ for case 3. This behavior indicates the dependence of the Poisson’s ratio of the BNNSs on the values of the input parameters, rather than being dependent on the NS anisotropy.

**Figure 13:**
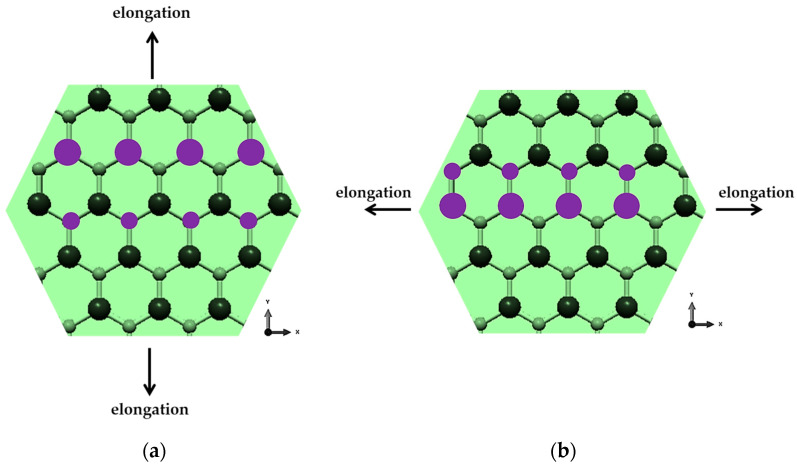
The behavior of the hexagonal BNNS lattices under axial loading in (**a**) zigzag and (**b**) armchair directions.

The difference between the Young’s moduli ($E_{x}$ > $E_{y}$) of the BNNSs can be explained by dissimilar stresses necessary for the contraction of the hexagonal NSs in the zigzag and armchair directions, which occur under longitudinal and transversal loads, respectively, as schematically illustrated in Figure 13.

When the NS is stretching along the *x*-axis (zigzag orientation), the contraction occurs by displacements of the two hexagonal lattice nodes (corresponding to the connected points of beam elements in the present model) in each cell (Figure 13a). In the case of the stretching along the *y*-axis (armchair orientation), four nodes in each hexagon cell are shifted to compress the NS (Figure 13b). Note that the behavior of Poisson’s ratios can be explained by the scheme shown in Figure 13 only for case 1 of the input parameters, for which $\nu_{\mathrm{xy}}$/$\nu_{\mathrm{yx}}$ = 1.22. For case 2 and 3, $\nu_{\mathrm{xy}}$/$\nu_{\mathrm{yx}}$ = 0.55 and 0.95, respectively, which indicates that $\nu_{\mathrm{xy}}$ < $\nu_{\mathrm{yx}}$ and the difference between the Poisson’s ratios evaluated, when a force is applied in the longitudinal and the transversal directions, is defined by the input parameters.

To facilitate understanding, the results from Figure 7, Figure 8, Figure 9, Figure 10, Figure 11 and Figure 12 are summarized in Table 4.

### 3.3. Shear Moduli of BNNSs and BNNRs

**Figure 14:**
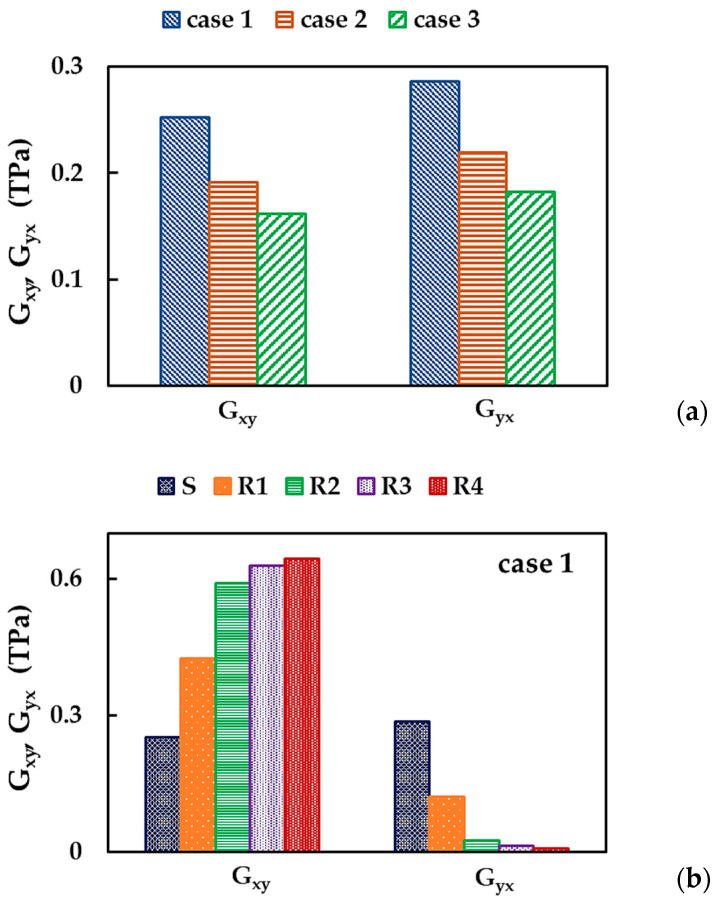
Shear moduli, $G_{\mathrm{xy}}$ and $G_{\mathrm{yx}}$, of (**a**) BNNSs for the three cases of the input parameters; and (**b**) BNNRs and BNNS for case 1.

Figure 14a shows the BNNSs shear moduli, $G_{\mathrm{xy}}$ and $G_{\mathrm{yx}}$, evaluated by the respective Equations (14) and (15), for the three cases of the input parameters. The value of $G_{\mathrm{xy}}$ (see Figure 4c) calculated for case 1 is 32% and 56% higher than those for cases 2 and 3, respectively. The shear modulus, $G_{\mathrm{yx}}$ (see Figure 4d), for case 1 is 31% and 38% higher when compared to $G_{\mathrm{yx}}$ for cases 2 and 3, respectively. The shear moduli for the BN nanoribbons and nanosheets are presented in Figure 14b for case 1. The $G_{\mathrm{xy}}$ modulus increases and $G_{\mathrm{yx}}$ decreases with the increase of the NS aspect ratio.

**Figure 15:**
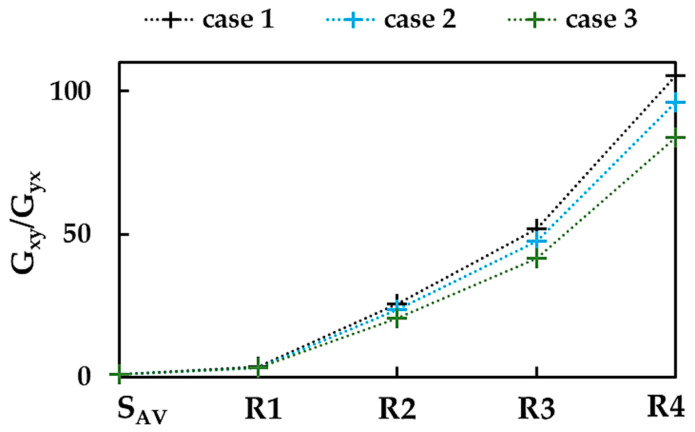
Evolutions of the ratio $G_{\mathrm{xy}}$/$G_{\mathrm{yx}}$ for BNNSs and BNNRs, with the NS aspect ratio.

Figure 14a,b suggests that different mechanical response for NS (NR) configurations was also observed for in-plane shear loading. To investigate this anisotropic behavior, the ratio between shear moduli, $G_{\mathrm{xy}}$/$G_{\mathrm{yx}}$ was plotted for BNNSs and BNNRs in Figure 15, considering the three cases of the input parameters.

The shear modulus, $G_{\mathrm{xy}}$, is 12% lower than the shear modulus, $G_{\mathrm{yx}}$. The value of $G_{\mathrm{xy}}$/$G_{\mathrm{yx}}$ increases with increasing aspect ratio, i.e., from the nanosheet to the R4 nanoribbon, in the cases 1, 2, and 3. The $G_{\mathrm{xy}}$/$G_{\mathrm{yx}}$ is ≈0.88 for the BNNSs for cases 1, 2, and 3; however, for the BNNRs, the $G_{\mathrm{xy}}$/$G_{\mathrm{yx}}$ ratio becomes sensitive to the input parameters and increases up to 105.6, 96.2, and 83.8 for cases 1, 2, and 3, respectively. For the BNNRs, starting with nanoribbon R2 ($L_{y}{:L}_{x}$ = 1:5), the values of the ratio between shear moduli for both orientations evaluated for case 1 (UFF) are about 9% and 23% higher than those for cases 2 (DFT + MM) and 3 (DFT), respectively.

**Figure 16:**
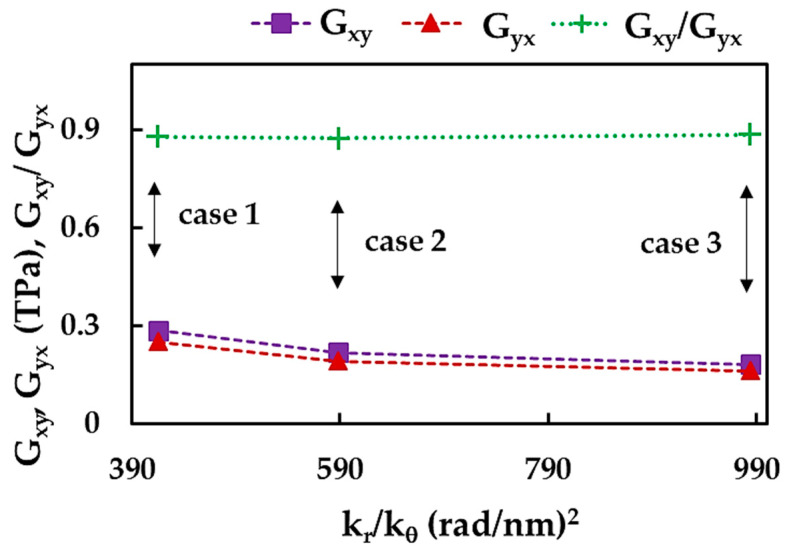
Evolutions of the BNNS shear moduli, $G_{\mathrm{xy}}$ and $G_{\mathrm{yx}}$, and their relationship, $G_{\mathrm{xy}}$/$G_{\mathrm{yx}}$, with the ratio of the bond stretching and bond bending force constants, $k_{r}$/$k_{\theta }$.

To study the influence of the input parameters on the results of the BNNS shear moduli, $G_{\mathrm{xy}}$, $G_{\mathrm{yx}}$ and $G_{\mathrm{xy}}$/$G_{\mathrm{yx}}$ were plotted as a function of the ratio between the force constants, $k_{r}$/$k_{\theta }$, in Figure 16. Both shear moduli, $G_{\mathrm{xy}}$, and $G_{\mathrm{yx}}$, decreases with the increase of the $k_{r}$/$k_{\theta }$ ratio, and the difference between $G_{\mathrm{xy}}$ and $G_{\mathrm{yx}}$ does not exceed ≈20% when moving from case 2 to 3. The relationship between the two shear moduli, $G_{\mathrm{xy}}$/$G_{\mathrm{yx}}$, is nearly independent of the case of the input parameter.

The results shown in Figure 16 can be better understood by analyzing the bending force constant, $k_{\theta }$. Indeed, the shear moduli, $G_{\mathrm{xy}}$ and $G_{\mathrm{yx}}$, decrease from case 1 to case 3, with a decreasing value of $k_{\theta }$, $k_{\theta }^{1}>k_{\theta }^{2}>k_{\theta }^{3}$ (see, Table 2).

**Figure 17:**
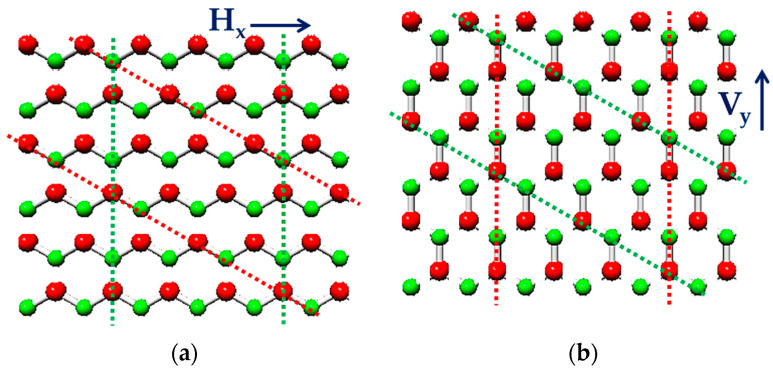
Exemplification of the shear behavior of BNNSs in the plane of hexagonal lattices under (**a**) horizontal loading and (**b**) vertical loading. The B atoms are shown in red; the N atoms are in green. The dashed lines are plotted to facilitate the perception of the relative atomic positions.

The mild NS shear modulus anisotropy can be explained by the orientation of the hexagonal BN lattice with respect to the directions of applied horizontal, $H_{x}$, or vertical, $V_{y}$, in-plane shear force, as shown in Figure 17. Under shear load $H_{x}$, the atoms to be moved for shear deformation to occur are connected by bonds, which are not aligned with the force direction, as exemplified in Figure 17a. When the force $V_{y}$ is applied to deform the BNNS, it is necessary to displace atoms with bonds between them, parallel to the load direction (see, Figure 17b).

To simplify understanding, the shear modulus results shown in Figure 14, Figure 15 and Figure 16 for the BNNSs and BNNRs are summarised in Table 5.

### 3.4. Comparison with the Literature Results

**Figure 18:**
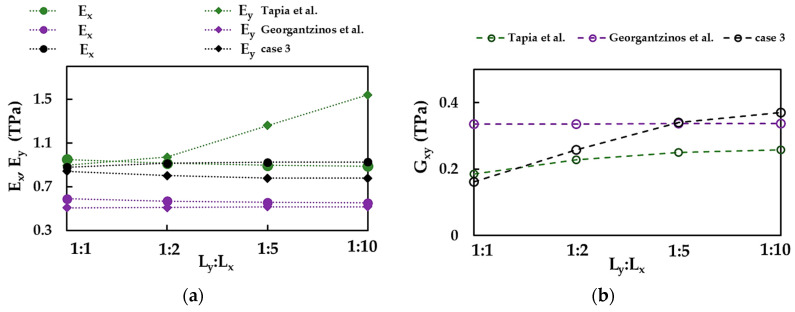
Comparison of the current values of (**a**) Young’s moduli, $E_{x}$ and $E_{y}$, and (**b**) shear modulus, $G_{\mathrm{xy}}$, for case 3, with those by Georgantzinos et al. [38] and Tapia et al. [39].

First of all, the current Young’s, $E_{x}$ and $E_{y}$, and shear, $G_{\mathrm{xy}}$, moduli of the BNNSs and BNNRs were compared with those available in the literature as shown in Figure 18a,b. Despite the differences between the Young’s modulus values, their evolutions with the NS aspect ratio show similar behavior, except $E_{y}$ reported by Tapia et al. [39] (see, Figure 18a). Similar to the current $G_{\mathrm{xy}}$, the shear modulus evaluated by Tapia et al. [39] increases with increasing the NS width, $L_{x}$, although at a slower rate. The value of $G_{\mathrm{xy}}$ reported by Georgantzinos et al. [38] is nearly constant, regardless of the NS aspect ratio.

Table 6 summarizes the current results on the elastic properties of square BNNSs and those available in the literature, including theoretical (numerical and analytical) and experimental results.

**Figure 19:**
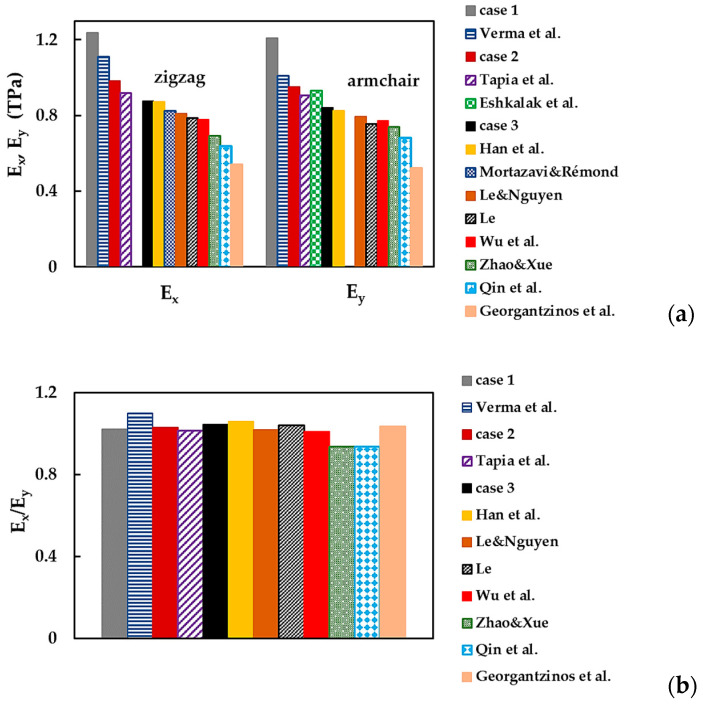
(**a**) Young’s moduli, $E_{x}$ and $E_{y}$, and (**b**) the ratio, $E_{x}$/$E_{y}$, of the BNNSs, obtained in the current study and reported by the other authors [22,26,27,28,29,33,34,37,38,39,40] (see Table 6).

To simplify the comparison of the current results and those available in the literature (see Table 6); the Young’s moduli, $E_{x}$ and $E_{y}$, and their relationship, $E_{x}$/$E_{y}$, are presented respectively in Figure 19a,b.

In Figure 19a, the Young’s moduli, $E_{x}$ and $E_{y}$, are arranged in descending order, from the values obtained for case 1 (UFF) to those by Georgantzinos et al. [38], who employed the NCM/MSM approach with spring elements. The $E_{x}$ and $E_{y}$ moduli evaluated for cases 2 (DFT + MM) and 3 (DFT) of the input parameters showed satisfactory agreement (difference in the range of 2.5% to 13.7%) with most of the previously reported $E_{x}$ and $E_{y}$ values [22,26,27,29,33,34,37,39]. The best agreement was observed when the $E_{x}$ and $E_{y}$ moduli, obtained for case 3, were compared with those evaluated by Han et al. [28], with a difference of 0.4% and 1.8%, respectively. Note that the use of numerical simulation input parameters computed by resourcing to the UFF method (case 1) led to overestimated values of the Young’s moduli, $E_{x}$ and $E_{y}$, compared with those presented in Figure 19a.

The results shown in Figure 19a suggest that the BNNSs have an anisotropic behavior, which can be expressed by the ratio between the Young’s moduli, $E_{x}/E_{y}$. The values of this ratio presented in Figure 19b indicate a mild anisotropy of the BN nanosheets. As in the present study, most authors found that the Young’s modulus $E_{x}$ is bigger than $E_{y}$ ($E_{x}$ > $E_{y}$), and the $E_{x}/E_{y}$ ratio was evaluated in the range of 1.01 to 1.04. Among the values presented in Figure 19b, Han et al. [28] and Verma et al. [29] reported the highest anisotropy ratios, $E_{x}/E_{y}$ ≈ 1.06 and 1.10, respectively. However, Zhao and Xue [27] and Qin et al. [40] determined that the Young’s modulus $E_{x}$ is smaller than $E_{y}$, with a ratio, $E_{x}/E_{y}$ ≈ 0.94, in both studies.

**Figure 20:**
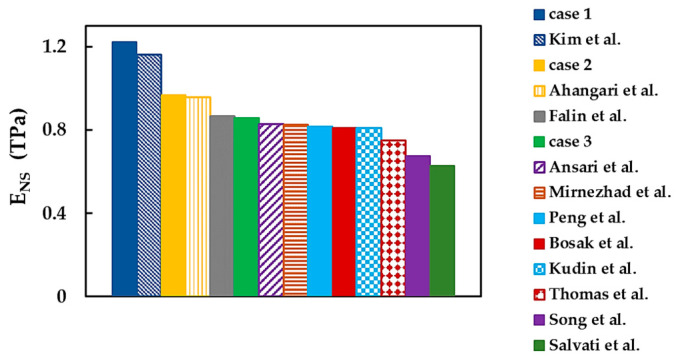
Comparison of the current Young’s modulus, $E_{\mathrm{NS}}$, with those from the literature [6,14,21,23,24,25,31,32,41,42,43].

Figure 20 compares the Young’s modulus results from the works presented in Table 6, including experimental studies—in which only one Young’s modulus was available—with the current average value assessed by $E_{\mathrm{NS}}=\left(E_{x}+E_{y}\right)/2$.

The value of $E_{\mathrm{NS}}$ calculated for case 1 shows a reasonable concordance (difference of 5.4%) with that obtained by Kim et al. [43] using the AFM nanoindentation test. The average Young’s modulus for case 2 is in good agreement (difference of 1.2%) with that of Ahangari et al. [23], who employed ab initio DFT calculations. The $E_{\mathrm{NS}}$ moduli observed for case 3 are comparable to those in the experimental studies by Falin et al. [6] and Bosak et al. [42], as well as the theoretical results by Kudin et al. [21], Peng et al. [24], Mirnezhad et al. [25], and Ansari et al. [41] (see Figure 20 and Table 6). It can be concluded that, essentially, the current Young’s modulus results are in adequate agreement with those in the literature, including experimental values. Better concordance is observed when literature values are compared with those obtained for cases 2 (DFT + MM) and 3 (DFT) of the input parameters.

**Figure 21:**
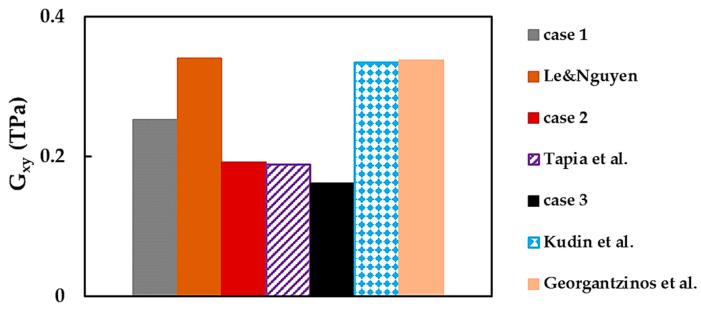
Comparison of the current shear modulus, $G_{\mathrm{xy}}$, and the values reported by other authors [21,37,38,39].

As seen in Table 6, the results of the BNNS shear modulus are infrequent in the literature. The comparison of the current shear modulus, $G_{\mathrm{xy}}$, with those reported by other authors is shown in Figure 21, where the scattering of the results is noticeable. The value of $G_{\mathrm{xy}}$ assessed for case 2 is in good agreement (the difference of 1.9%) with that evaluated by Tapia et al. [39], whose study shares the same modeling approach—NCM/MSM, employing beams—with the current work.

In Figure 21, the BNNS shear modulus evaluated by Kudin et al. [21], Le and Nguyen [37], and Georgantzinos et al. [38] have comparable values of about 0.339 TPa, despite dissimilar modeling approaches used—ab initio DFT [21], NCM/MSM employing 2-node stretching and 3-node angle bending [37], and spring elements [38]—to represent B–N bond. The value of $G_{\mathrm{xy}}$ assessed in the abovementioned studies are higher than those of the current work (cases 1, 2, and 3) and Tapia et al. [39]. This smoother shear response and lower value of $G_{\mathrm{xy}}$, observed in the current work and in that of Tapia et al. [39], can be possibly justified by the elastic element formulation, i.e., beam elements were used for modeling the B–N bond in both studies. The 2-node stretching and 3-node angle bending elements, which form the global stiffness matrix and the springs utilized in the works of Le and Nguyen [37] and Georgantzinos et al. [38], respectively, have a smaller number of degrees of freedom and consequently are stiffer. This may be a conceivable explanation for the higher shear modulus values found in the abovementioned studies [37,38]. To the best of our knowledge, among the existing works, only Georgantzinos et al. [38] reported the shear modulus for both orientations, $G_{\mathrm{xy}}$ and $G_{\mathrm{yx}}$,. Their ratio is about 0.98, which is higher than the current ratio $G_{\mathrm{xy}}$/$G_{\mathrm{yx}}$ ≈ 0.88.

The values of the Poisson’s ratio, $\nu_{\mathrm{xy}}$, in Table 6 show considerable scattering in the range of 0.211 [21] to 0.822 [39]. Among the studies mentioned in Table 6, Le and Nguyen [37] and Georgantzinos et al. [38], in addition to the $\nu_{\mathrm{xy}}$ value, reported another Poisson’s ratio, $\nu_{\mathrm{yx}}$, with a ratio $\nu_{\mathrm{xy}}/\nu_{\mathrm{yx}}$ ≈ 1.018 and 1.005, respectively. These values indicate that $\nu_{\mathrm{xy}}$ is slightly higher than $\nu_{\mathrm{yx}}$, unlike the current study in which $\nu_{\mathrm{xy}}/\nu_{\mathrm{yx}}$ is 1.22 (case 1), 0.55 (case 2), and 0.95 (case 3). It is worth noting that the values of $\nu_{\mathrm{xy}}$ and $\nu_{\mathrm{yx}}$, assessed by Georgantzinos et al. [38], are averages obtained for square and rectangular BNNSs in a wide range of their aspect ratio.

## 4. Conclusions

The elastic properties of squared and rectangular (viz. nanoribbons) boron nitride nanosheets with various sizes and aspect ratios were evaluated using numerical simulation based on the NCM/MSM approach. The present study provides a robust finite element model of the square and rectangular BNNSs, which allows rapid and reliable determination of their Young’s and shear moduli and Poisson’s ratio.

The values of the Young’s and shear moduli and the Poisson’s ratio of BNNSs are influenced by the aspect ratio of the nanosheet and nearly independent of the nanosheet size. These three elastic constants are sensitive to the loading case and their influence increases with the nanosheet aspect ratio.

Three sets of input parameters were used for the numerical simulation and the sensitivity of the elastic properties of the square and rectangular BNNSs to the chosen set was analyzed. The input parameters calculated by the UFF method led to the highest values of the Young’s and shear moduli. On the contrary, the input parameters calculated using the DFT + MM approach and those based on direct DFT calculations provided the Young’s modulus results, which are in satisfactory agreement with those reported in the literature.

The current Young’s modulus values show good concordance with experimental ones.

Knowledge of the elastic properties permits envisaging the capacity of 2D boron nitride nanostructures to reinforce composites and their effectiveness in strain engineering applications.

The results establish a benchmark for evaluating the elastic properties of boron nitride nanosheets by theoretical methods.

## Figures and Tables

**Table 1:** The geometry of the studied single-layer boron nitride NSs and NRs.

Designation	L_x_, nm	L_y_, nm	Aspect Ratio, $L_{y}{:L}_{x}$	Number of Elements	Number of Nodes
S1	2.04	2.06	1:1	237	170
S2	4.07	4.26	1:1	954	660
S3	10.18	9.98	1:1	5503	3726
S4	14.26	13.94	1:1	10,728	7232
R1	4.07	2.06	1:2	469	330
R2	10.18	2.06	1:5	1165	810
R3	14.26	2.06	1:7	1629	1130
R4	20.37	2.06	1:10	2325	1610

**Table 2:** Input parameters for numerical simulations of square and rectangular BN nanosheets: geometrical and mechanical properties of the beam elements, for the three cases considered.

Case	Method	Force Field Constants	*l*, nm	d, nm	E_b_, GPa	G_b_, GPa	ν_b_
1	^1^ UFF	$k_{r}$ = 676 nN/nm $k_{\theta }$ = 1.627 nN·nm/rad^2^ ^2^ $k_{\tau }$ = 2.470 nN·nm/rad^2^	0.145	0.1962	3243	2462	0.10
2	^1^ DFT + MM	$k_{r}$ = 585 nN/nm $k_{\theta }$ = 0.994 nN·nm/rad^2^ ^2^ $k_{\tau }$ = 2.470 nN·nm/rad^2^		0.1648	3977	4941	0.21
3	^3^ DFT	$k_{r}$ = 617 nN/nm $k_{\theta }$ = 0.627 nN·nm/rad^2^ $k_{\tau }$ = 0.132 nN·nm/rad^2^	1.447	0.1275	6989	737	0.38

^1^ $k_{r}$ and $k_{\theta }$ calculated in the current study, based on the respective method; ^2^ k_τ_ obtained from the work of Ansari et al. [49]; ^3^ *l*, $k_{r}$, $k_{\theta }$, and k_τ_ obtained from the work of Tapia et al. [39].

**Table 3:** Average values of the Young’s and shear moduli, and the Poisson’s ratio of the BNNSs nanosheets of different sizes, for the three cases in Table 2.

Case	$E_{x}$, TPa	$E_{y}$, TPa	$G_{\mathrm{xy}}$, TPa	$G_{\mathrm{yx}}$, TPa	$\nu_{\mathrm{xy}}$	$\nu_{\mathrm{yx}}$
1	1.237	1.209	0.252	0.286	0.033	0.027
2	0.982	0.953	0.191	0.219	0.007	0.013
3	0.877	0.840	0.162	0.182	0.079	0.083

**Table 4:** Results of the Young’s moduli and Poisson’s ratio for the BN nanosheets and nanoribbons.

NS/NR	Case	$E_{x}$, TPa	$E_{y}$, TPa	$E_{x}$ $/E_{y}$	$\nu_{\mathrm{xy}}$	$\nu_{\mathrm{yx}}$	$\nu_{\mathrm{xy}}$ $/\nu_{\mathrm{yx}}$
S_AV_	1	1.237	1.209	1.02	0.033	0.027	1.22
	2	0.982	0.953	1.03	0.007	0.013	0.55
	3	0.877	0.840	1.04	0.079	0.083	0.95
R1	1	1.282	1.207	1.06	0.036	0.020	1.84
	2	1.021	0.942	1.08	0.007	0.012	0.58
	3	0.913	0.802	1.14	0.085	0.067	1.26
R2	1	1.290	1.212	1.06	0.036	0.011	3.30
	2	1.029	0.938	1.10	0.008	0.006	1.36
	3	0.922	0.778	1.18	0.086	0.035	2.45
R3	1	1.291	1.213	1.06	0.036	0.008	4.58
	2	1.030	0.938	1.10	0.008	0.004	1.90
	3	0.923	0.777	1.19	0.086	0.025	3.39
R4	1	1.293	1.213	1.07	0.036	0.005	6.55
	2	1.032	0.938	1.10	0.008	0.003	2.70
	3	0.925	0.777	1.19	0.086	0.018	4.84

**Table 5:** Shear modulus results for BN nanosheets and nanoribbons.

NS/NR	Case	$G_{\mathrm{xy}}$, TPa	$G_{\mathrm{yx}}$, TPa	$G_{\mathrm{xy}}$ $/G_{\mathrm{yx}}$
S_AV_	1	0.252	0.286	0.88
	2	0.191	0.219	0.87
	3	0.162	0.182	0.89
R1	1	0.425	0.120	3.55
	2	0.318	0.094	3.39
	3	0.258	0.082	3.15
R2	1	0.590	0.023	25.4
	2	0.433	0.018	23.4
	3	0.340	0.016	20.6
R3	1	0.628	0.012	51.8
	2	0.460	0.010	47.5
	3	0.360	0.009	41.6
R4	1	0.643	0.006	105.6
	2	0.472	0.005	96.2
	3	0.370	0.004	83.8

**Table 6:** Comparison of the results of current Young’s and shear moduli, and Poisson’s ratio for boron nitride nanosheets with those reported in the literature.

Reference	Method	$t_{n}$, nm	$E_{x}$, TPa	$E_{y}$, TPa	$E_{x}$ $/E_{y}$	$G_{\mathrm{xy}}$, TPa	$\nu_{\mathrm{xy}}$ $,\;\nu_{\mathrm{yx}}$	Size, nm^2^
	Atomistic approach:							
Kudin et al. [21]	ab initio DFT	0.335	0.810		–	0.334	0.211	–
Wu et al. [22]		0.330	0.780	0.773	1.01	–	–	1.500 × 1.732
Ahangari et al. [23]		0.320	0.956		–	–	–	1.005 × 1.132
Peng et al. [24]		–	0.818 ^1^		–	–	–	–
Mirnezhad et al. [25]	DFT + QHA	–	0.825 ^1^		–	–	–	–
Zhao and Xue [27]	MD: Tersoff potential	0.330	0.692	0.739	0.94	–	–	12.00 × 12.00
Thomas et al. [31]		0.334	0.750		–	–	0.297	10,000 atoms
Eshkalak et al. [33]		0.340	–	0.930	–	–	–	5.30 × 5.60
Mortazavi and Rémond [34]		0.330	0.824	–	–	–	–	–
Le [26]	MD: Tersoff and Tersoff-like potentials	0.335	0.786	0.756	1.04	–	–	10.36 × 10.22
Han et al. [28]	MD: Tersoff-like potential	0.333	0.874	0.825	1.06		–	10.00 × 10.00
Verma et al. [29]	MD: Tersoff-Berner potential	0.333	1.110	1.010	1.10	–	–	8.67 × 10.22
Salvati et al. [32]	MD: modified Tersoff potential	0.340	0.628		–	–	–	30.00 × 30.00
	NCM/MSM approach:							
Le and Nguyen [37]	2-node stretching and 3-node angle bending elements	0.335	0.809	0.794	1.02	0.340	0.226; 0.222	10.42 × 10.29
Ansari et al. [41]	analytical solution	0.340	0.829		–	–	–	–
Georgantzinos et al. [38]	springs	0.340	0.540	0.522	1.03	0.338; 0.346 ^2^	0.420; 0.418	from 2.00 × 2.00 to 20.00 × 20.00
Tapia et al. [39]	beams	0.106	0.920	0.908	1.01	0.188	0.822	3.76 × 3.76 7.27 × 7.24 14.79 × 14.71
Qin et al. [40]		–	0.637 ^1^	0.681 ^1^	0.94	–	–	9.00 × 9.00
Current study		0.340	1.237	1.209	1.02	0.252; 0.286 ^2^	0.033; 0.027	2.04 × 2.06 4.07 × 4.26 10.18 × 9.98 14.26 × 13.94
			0.982	0.953	1.03	0.191; 0.219 ^2^	0.007; 0.013	
			0.877	0.840	1.04	0.162; 0.182 ^2^	0.079; 0.083	
	Experimental:							
Bosak et al. [42]	X-ray scattering measurements	–	0.811		–	–	–	–
Song et al. [14]	nanoindentation + AFM	0.330	0.676		–	–	–	7.85 × 10^4^ (circular)
Falin et al. [6]	nanoindentation	0.334	0.865 ± 0.073		–	–	–	–
Kim et al. [43]	nanoindentation + AFM	–	1.16 ± 0.1		–	–	–	–

^1^ Calculated from the surface Young’s modulus, $E_{\mathrm{sNS}}$, by $E_{\mathrm{NS}}=E_{\mathrm{sNS}}{/t}_{n}$, for NS thickness $t_{n}$ = 0.34 nm; ^2^ $G_{\mathrm{yx}}$.

## Data Availability

The data presented in this study are available on request from the corresponding author after obtaining permission from an authorized person.
